# Change in HIV‐related characteristics of children hospitalised with infectious diseases in Western Cape, South Africa, 2008–2021: a time trend analysis

**DOI:** 10.1002/jia2.26151

**Published:** 2023-11-01

**Authors:** Shani T. de Beer, Amy L. Slogrove, Brian Eley, Suzanne M. Ingle, Hayley E. Jones, Florence Phelanyane, Kim Anderson, Emma Kalk, Andrew Boulle, Mary‐Ann Davies

**Affiliations:** ^1^ Centre for Infectious Disease Epidemiology and Research School of Public Health University of Cape Town Cape Town South Africa; ^2^ Health Intelligence Directorate Western Cape Government Health Cape Town South Africa; ^3^ Population Health Sciences Bristol Medical School University of Bristol Bristol UK; ^4^ Department of Paediatrics & Child Health Faculty of Medicine & Health Sciences, Stellenbosch University Worcester South Africa; ^5^ Paediatric Infectious Diseases Unit Red Cross War Memorial Children's Hospital and the Department of Paediatrics and Child Health University of Cape Town Cape Town South Africa; ^6^ Division of Public Health Medicine School of Public Health University of Cape Town Cape Town South Africa

**Keywords:** HIV exposure, HEU, hospitalisation, infectious disease, vertical HIV transmission prevention, South Africa

## Abstract

**Introduction:**

With the scaling up of vertical HIV transmission prevention programmes, the HIV‐related population profile of children in South Africa has shifted. We described temporal changes in HIV‐related characteristics of children, aged ≤3 years (up to the third birthday), with infectious disease hospitalisations across the Western Cape province.

**Methods:**

We used routinely collected electronic data to identify children born in the Western Cape with infectious disease hospital records for lower respiratory tract infections, diarrhoea, meningitis and tuberculous meningitis, from 2008 to 2021. Linked maternal and child unique identifiers were used to extract pregnancy, HIV‐related, laboratory, pharmacy and hospitalisation data. We described temporal changes in child HIV exposure and acquisition status, timing of maternal HIV diagnosis and antiretroviral therapy (ART) start, infant exposure to maternal ART and timing thereof, and maternal CD4 and HIV viral load closest to delivery. We used logistic and multinomial regression to assess changes in characteristics between the Pre‐Option B+ (2008–2013), Option B+ (2013–2016) and Universal ART periods (2016–2021).

**Results:**

Among 52,811 children aged ≤3 years with hospitalisations, the proportion living with HIV dreased from 7.0% (2008) to 1.1% (2021), while those exposed to HIV and uninfected increased from 14.0% (2008) to 16.1% (2021) with a peak of 18.3% in 2017. Among mothers with HIV (*n* = 9873), the proportion diagnosed with HIV and starting ART before pregnancy increased from 20.2% to 69.2% and 5.8% to 59.0%, respectively, between 2008 and 2021. Children hospitalised during the Universal ART period had eight times higher odds (Odds Ratio: 8.41; 95% CI: 7.36–9.61) of exposure to maternal ART versus children admitted Pre‐Option B+. Among mothers of children exposed to HIV and uninfected with CD4 records (*n* = 7523), the proportion with CD4 <350 cells/μl decreased from 90.6% (2008) to 27.8% (2021).

**Conclusions:**

In recent years, among children hospitalised with infectious diseases, there were fewer children with perinatally acquired HIV, while an increased proportion of those without HIV acquisition are exposed to maternal HIV and ART. There is a need to look beyond paediatric HIV prevalence and consider child exposure to HIV and ART among children without HIV, when assessing the HIV epidemic's impact on child health services.

## 1 INTRODUCTION

Infectious diseases are a leading cause of paediatric morbidity and mortality and place a burden on public healthcare services, particularly those that are already stretched [1, 2, 3, 4]. Evidence suggests that both HIV acquisition and exposure without HIV acquisition are associated with infectious diseases. Between 1992 and 1997, HIV prevalence among paediatric admissions at an urban hospital in South Africa (SA) increased from 2.9% to 20.0%[5, 6], with a related increase in infectious diseases admissions. Furthermore, children exposed to HIV and uninfected (HEU) have been reported to have a higher risk of infection‐related morbidity than children unexposed to HIV and uninfected (HUU) [7, 8].

The last two decades have seen substantial scale‐up and success of vertical HIV transmission prevention (VTP) in SA [9]. This success was largely attributable to increased access to antiretroviral therapy (ART) for pregnant people living with HIV, with improved and simplified guideline recommendations. In 2004, ART was only recommended in pregnancy for those with CD4 count <200 cells/μl; in 2008, ART was expanded to those with CD4 <350 cells/μl [9, 10]. Only in 2015 (2013 in the Western Cape province of SA) did ART become available to all pregnant and breastfeeding persons living with HIV, regardless of CD4 count (“Option B+”) [11]. In 2016, ART became universally available to all persons living with HIV, meaning more of them would have access to ART before their first pregnancy [12]. Consequently, SA has seen a shift in the HIV‐related profile of children under the age 15 years; between 2000 and 2018 HIV prevalence in children decreased by 74.0%, while the proportion who are HEU increased over seven‐fold, accounting for 21.6% of all children (age 0–14 years) in SA in 2018 [13, 14]. Furthermore, ART coverage in pregnancy in SA was 96.0% in 2019 [15, 16], meaning that most children HEU are exposed to ART during gestation. Studies have previously used HIV prevalence among hospitalised children as an indicator of the HIV epidemic's impact on child health services [6, 17]. However, with the growing population of children HEU, and evidence that in utero HIV and ART exposure are also associated with child health outcomes in the absence of child HIV acquisition [13], it is also important to measure these characteristics, to more comprehensively understand the effect of the HIV epidemic on child health services.

We aimed to describe, at a provincial‐level, the temporal changes in HIV‐related characteristics of children hospitalised with infectious diseases from 2008 to 2021, using longitudinal individual‐level routine care data from the Western Cape Provincial Health Data Centre.

## 2 METHODS

### 2.1 Study design and data source

This retrospective, population‐based analysis used digitised routine maternal and child healthcare data from the Western Cape Provincial Health Data Centre. The data centre is an electronic health information platform that uses a unique health identifier to consolidate multiple sources of individual‐level data from provincial public sector health services in the Western Cape [18]. Data including hospital admissions, outpatient visits, laboratory test results and pharmacy records are linked into a single individual‐level data repository. Data on mothers and children are also electronically linked.

### 2.2 Study setting

The Western Cape, one of SA's nine provinces, has an estimated population of seven million [18]. The province's antenatal HIV prevalence increased from 16.1% (2008) to 17.9% (2019) [15]. About three‐quarters of the population, including most persons living with HIV, utilise public‐sector health services [18, 19]. The public sector includes 51 hospitals consisting of District, Regional, Tertiary and Central hospitals [20]. District hospitals are the usual entry point into the hospital system, with complicated cases being referred to Regional, Tertiary or Central hospitals for specialist care [21]. In the late 1990s and early 2000s, ill children, particularly children with HIV (CWH) or children HEU, were admitted to Tertiary/Central hospitals. However, over time, there have been changes to paediatric healthcare organisation in SA, with increased district hospital capacity and less dependence on tertiary hospitals [22, 23].

### 2.3 Study participants

We used routinely collected electronic data to identify children born in the Western Cape between 2008 and 2018, with a known live birth outcome, who had infectious disease hospitalisation records for lower respiratory tract infection (LRTI) (including influenza, viral, bacterial and congenital pneumonias, and bronchitis), diarrhoea, meningitis or tuberculous meningitis (TBM), from 2008 to 2021 (Supplementary Figure 1). We included first admissions in children hospitalised aged ≤3 years (by their third birthday). Within the Western Cape Provincial Health Data Centre, hospital admissions are classified using ICD‐10 codes. Admissions with more than one of the above infectious diagnoses were counted in each relevant category.

To accurately classify child HIV acquisition and in utero HIV exposure, children were excluded if they could not be linked to their mothers, if child HIV‐positive status was confirmed after hospital discharge but with unknown timing of acquisition, if their mothers were diagnosed with HIV more than 10 weeks after child's date of birth (10 weeks postnatal was used as a reasonable time‐period to consider that maternal HIV acquisition might have occurred during pregnancy) or if they or their mothers had data inconsistencies (e.g. a negative HIV‐PCR result after evidence of having HIV).

### 2.4 Procedures and measurements

We extracted data on demographics, pregnancy, HIV testing, HIV evidence, ART start and dispensary dates, laboratory test results (HIV PCR and ELISA, CD4 count and viral load) and death from the Western Cape Provincial Health Data Centre. Using HIV‐related data, including data on most recent negative HIV test results relative to the time of hospital admission, we categorised the in utero HIV exposure status and HIV acquisition status of each child at infectious disease hospitalisation discharge date as CWH, HEU or HUU, according to simplified DECIPHER (Data Evaluation and Collaborative Initiation for Paediatric HIV Education and Research) definitions (Table S1) [24]. Children who tested positive were considered HIV negative until their last negative HIV test and to have an unknown HIV status thereafter until the date of first HIV evidence. CWH whose mothers were not known to be living with HIV were included for assessing change in HIV acquisition status over time, but excluded for the analysis of other HIV‐related characteristics.

For mothers with HIV, we categorised timing of maternal HIV diagnosis (before pregnancy, during pregnancy, at delivery/postnatally); timing of maternal ART start (before pregnancy, during pregnancy, delivery/postnatally, no ART evidence); infant exposure to maternal ART (yes/no) by using ART dispensing dates to determine if mothers were on ART at any point between conception and 3 months post‐delivery; timing of earliest infant exposure to maternal ART (at conception, early/middle gestation [post‐conception to 3 months pre‐delivery], late gestation/postnatally [within 3 months pre or post‐delivery]) based on the earliest point mothers were dispensed ART between conception and 3 months post‐delivery; maternal CD4 count (<350 cells/μl, 350–499 cells/μl, ≥500 cells/μl) and viral load (<1000 copies/ml, ≥1000 copies/ml), using records closest to delivery, within a 365‐day window of delivery. We described these characteristics over time by year of infectious disease hospital admission. We categorised the time of hospital admission into periods: January 2008–April 2013 (Pre‐Option B+), May 2013–August 2016 (Option B+) and September 2016–December 2021 (Universal ART).

### 2.5 Statistical analyses

We described and assessed differences in non‐HIV‐related child and maternal characteristics, by categorised year of admission, using proportions (categorical variables) and means or medians (continuous variables). We plotted trends in the proportions of different HIV‐specific characteristics among hospitalised children. Statistical evidence for changes in proportions across the three time periods was assessed using univariable logistic and multinomial regression models, for binary and non‐binary categorical variables, respectively, with the HIV‐related characteristic as the dependent variable and time (in periods) as the independent variable. All statistical analyses were done using STATA 17.0 [25, 26].

### 2.6 Ethics

This analysis was approved by the University of Cape Town Human Research Ethics Committee (REF 101/2021).

A waiver of informed consent was obtained for this research as all data had already been routinely collected by health services and no participant recruitment was required.

## 3 RESULTS

Between 2008 and 2018, there were 54,181 children (born at Western Cape public health facilities) who had a hospital admission record for at least one of the four infectious diseases of interest by age 3 years, of whom 52,811 (97.5%) were included in our analysis (Figure S1). Of the 1370 children who were excluded from our analysis, 70% had mothers diagnosed with HIV postnatally. The main difference between them and children included in the analysis was the proportion of child deaths by age 3 (2.4% vs. 1.5%) and the proportion of maternal deaths by child age 3 (1.1% vs. 0.5%). Of the admissions, 64.9% included LRTI, 36.5% diarrhoea, 4.0% meningitis and 0.6% TBM. The number of annual admissions varied substantially during the study period, with 75.5% of all admissions occurring from 2015 onwards (Table S2).

### 3.1 Non‐HIV‐related characteristics of infants and mothers

Among children included, 56.4% were male and 1.5% died before the age 3 years (Table 1). The proportion of admitted children with very low (1000–1499 g) and low birthweight (1500–2499 g) decreased between the Pre‐Option B+, Option B+ and Universal ART periods from 8.3% to 4.8% to 3.2% and 23.3% to 19.0% to 17.0%, respectively. Mean maternal age at delivery remained constant at 27 years throughout, while the proportion of mothers who died by child age 3 years decreased from 1% Pre‐Option B+ to 0.5% during Option B+ and 0.4% during the Universal ART period.

**Table 1:** Characteristics of infants (and their mothers), born in Western Cape from 2008 to 2018, who had an infectious disease hospital admission (LRTI, diarrhoea, meningitis and TBM), by different ART policy periods (Pre‐Option B+, Option B+ and Universal ART)

Infectious disease hospitalisation				
Variable	Total *N* = 52,811 (100%)	Pre‐Option B+ (A) *n* = 7223 (13.7%)	Option B+ (B) *n* = 16,030 (30.4%)	Universal ART (C) *n* = 29,558 (56.0%)
**Infant Characteristics**				
Sex: *n* (%)				
Male	29,761 (56.4)	4052 (56.1)	9101 (56.8)	16,608 (56.2)
Missing	15 (0.03)	0 (0)	4 (0.02)	11 (0.03)
Birthweight (g): *n* (%)				
Foetal macrosomia (≥4000 g)	1799 (3.4)	233 (3.2)	528 (3.3)	1038 (3.5)
Normal (2500–3999 g)	37,830 (71.6)	4448 (61.6)	11,316 (70.6)	22,066 (74.7)
Low (1500–2499 g)	9759 (18.5)	1681 (23.3)	3042 (19.0)	5036 (17.0)
Very low (1000–1499 g)	2318 (4.4)	600 (8.3)	774 (4.8)	944 (3.2)
Extremely low (<1000 g)	926 (1.8)	251 (3.5)	330 (2.1)	345 (1.2)
Missing	179 (0.3)	10 (0.1)	40 (0.3)	129 (0.4)
Mean (95% CI)	2868 (2862; 2874)	2684 (2665; 2704)	2844 (2832; 2856)	2926 (2918; 2934)
Twins: *n* (%)				
	2252 (4.3)	525 (7.3)	716 (4.5)	1011 (3.4)
Died before 3 years of age (all‐cause): *n* (%)				
	808 (1.5)	276 (3.8)	242 (1.5)	290 (1.0)
Age (months) at first infectious‐cause hospitalisation				
Median (IQR)	7.27 (11.97)	4.73 (8.35)	5.85 (9.86)	9.01 (13.58)
Exposure and HIV acquisition status at time of hospital discharge: *n* (%)				
Children HEU	8969 (17.0)	1025 (14.2)	2802 (17.5)	5142 (17.4)
Children HUU	42,864 (81.2)	5899 (81.7)	12,942 (80.7)	24,023 (81.3)
Children with HIV	978 (1.9)	299 (4.1)	286 (1.8)	393 (1.3)

**Maternal Characteristics**				
Age (years) at delivery:				
Mean (95% CI)	27.20 (27.11; 27.22)	27.23 (27.09; 27.38)	27.09 (26.99; 27.19)	27.19 (27.12; 27.26)
Missing: *n* (%)	243 (0.5)	48 (0.7)	77 (0.5)	118 (0.4)
Maternal death by child age 3 years: *n* (%)				
	253 (0.5)	74 (1.0)	73 (0.5)	106 (0.4)

*Note*: Pre‐Option B+ (January 2008–April 2013); Option B+ (May 2013–August 2016); Universal ART (September 2016–December 2021).

Abbreviations: ART, antiretroviral therapy; CI, confidence interval; HEU, exposed to HIV and uninfected; HUU, unexposed to HIV and uninfected; IQR, interquartile range; LRTI, lower respiratory tract infection; TBM, tuberculous meningitis.

### 3.2 HIV‐related characteristics of infants and mothers

#### 3.2.1 Infant HIV exposure and HIV acquisition status

By hospital discharge, 17.0% of children were HEU, 81.2% HUU and 1.9% CWH (Table 1). The certainty of the combined HIV exposure and acquisition status of most (56.4%) children HEU was high, but low for most children HUU (92.8%) (Table S3). The proportion of CWH among infectious disease admissions decreased from 7.0% in 2008 to 1.1% in 2021. The proportion of children classified as HEU at hospital discharge increased from 14.1% in 2008 to 16.1% in 2021, with a peak of 18.3% in 2017 (Figure 1.1). Children hospitalised with infectious diseases were less likely to have HIV, compared to being HEU, during the Option B+ (Relative Risk Ratio [RRR]: 0.35; 95% CI: 0.29–0.42) and Universal ART (RRR: 0.26; 95% CI: 0.22–0.31) periods, relative to children admitted during the Pre‐Option B+ period (Figure 1.2).

**Figure 1:**
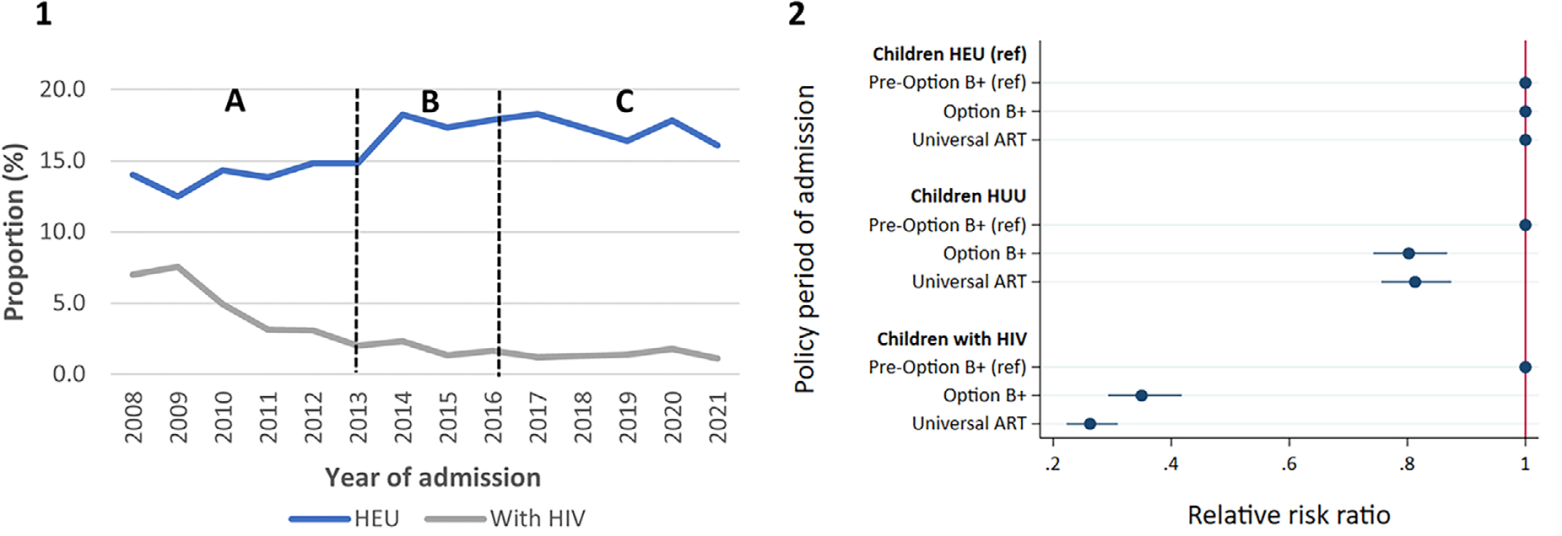
HIV exposure and aquisition status. [1]—Trends in HIV exposure and aquisition status among children HEU and CWH who were hospitalised with at least one of four infectious diseases (lower respiratory tract infection, diarrhoea, meningitis and tuberculous meningitis), by year. The vertical dotted lines demarcate different policy periods: A—Pre‐Option B+, B—Option B+, C—Universal ART; [2]—Plot of the relative risk ratios (with 95% confidence intervals) from multinomial logistic regressions assessing the association between child HIV exposure and aquisition status with policy period of hospital admission. *N* = 52,811. ART, antiretroviral therapy; CWH, children with HIV; HEU, exposed to HIV and uninfected; HUU, unexposed to HIV and uninfected; ref, reference group.

#### 3.2.2 Maternal HIV diagnosis and ART start time

Of CWH, 7.6% of mothers did not have evidence of HIV and were excluded from further analysis (Table S4). A descriptive summary of the proportion of mothers in respective maternal HIV diagnosis and ART start categories is shown in Table S5. Among mothers of hospitalised children HEU and CWH, who were living with HIV, the proportion diagnosed with HIV before pregnancy increased from 20.2% in 2008 to 69.2% in 2021, with a peak of 75.2% in 2020 (Figure 2.1). Mothers were more likely to have been diagnosed with HIV before pregnancy, versus during pregnancy, among children admitted during the Option B+ and Universal ART periods, compared to children admitted before the Option B+ period (Figure 2.2).

**Figure 2:**
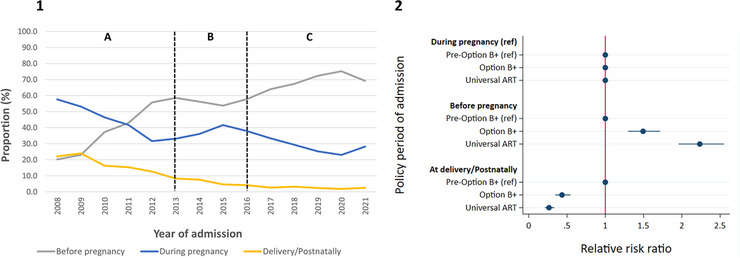
Timing of HIV diagnosis. [1]—Trends in timing of HIV diagnoses among mothers of children HEU and CWH, who were hospitalised with at least one of four infectious diseases (lower respiratory tract infection, diarrhoea, meningitis and tuberculous meningitis), by year. The vertical dotted lines demarcate different policy periods: A—Pre‐Option B+, B—Option B+, C—Universal ART; [2]—Plot of the relative risk ratios (with 95% confidence intervals) from multinomial logistic regression assessing the association of timing of mother HIV diagnosis (relative to pregnancy and delivery) with policy period of hospital admission. *N* = 9,873. ART, antiretroviral therapy; CWH, children with HIV; HEU, exposed to HIV and uninfected; ref, reference group.

The proportion of mothers with HIV (*N* = 9873) starting ART before pregnancy increased from 5.8% in 2008 to 59.0% in 2021, peaking at 62% in 2020 (Figure 3.1), with mothers more likely to have started ART before pregnancy, versus at delivery/postnatally, among children admitted during the Option B+ and Universal ART periods, compared to children admitted before the Option B+ period (Figure 3.2).

**Figure 3:**
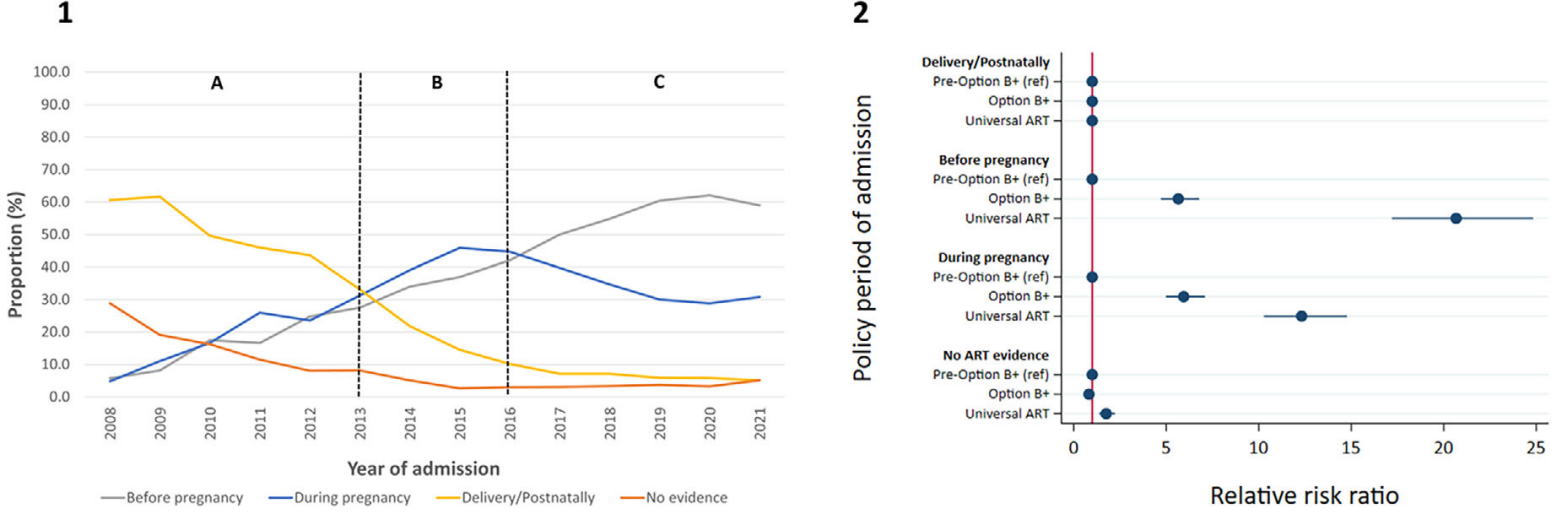
Timing of ART start. [1]—Trends in timing of ART start among mothers of children HEU and CWH who were hospitalised with at least one of four infectious diseases (lower respiratory tract infection, diarrhoea, meningitis and tuberculous meningitis), by year. The vertical dotted lines demarcate different policy periods: A—Pre‐Option B+, B—Option B+, C—Universal ART; [2]—Plot of the relative risk ratios (with 95% confidence intervals) from multinomial logistic regression assessing the association of timing of mother's ART start (relative to pregnancy and delivery) with policy period of hospital admission. *N* = 9873. ART, antiretroviral therapy; CWH, children with HIV; HEU, exposed to HIV and uninfected; ref, reference group.

### 3.3 Infant exposure to maternal ART

The proportion of children exposed to HIV (HEU and CWH) who were exposed to maternal ART increased from 16.3% in 2008 to 87.2% in 2021 (Figure S2.1). The odds of having been exposed to maternal ART increased eight‐fold (Odds Ratio: 8.41; 95% CI: 7.36–9.61) for children admitted to the hospital during the Universal ART period compared to children admitted before the Option B+ period (Figure S2.2).

Among children exposed to maternal ART, the proportion exposed for the first time during late gestation/postnatally decreased from 58.8% in 2008 to 17.6% in 2021 (Figure S3.1). Children admitted to hospital with an infectious disease during the Option B+ and Universal ART periods were more likely to have first been exposed to maternal ART at conception, relative to late gestation/postnatally, compared with children admitted before the Option B+ period) (Figure S3.2).

### 3.4 Maternal viral load and CD4 count closest to pregnancy start

Among hospitalised children HEU with mothers who had viral load tests, >80% of mothers had viral loads <1000 copies/ml for all years except 2010 (72.7%) (Figure 4.1). Mothers of children HEU were more likely to have viral loads <1000 copies/ml (vs. ≥1000 copies/ml) for children admitted during the Universal ART period, compared to the period Pre‐Option B+ (Figure 4.3). Among hospitalised CWH, the proportion of mothers with viral loads <1000 copies/ml peaked at 58.3% in 2009 and decreased to 25% in 2021 (Figure 4.2). Mothers of hospitalised CWH were less likely to have viral loads <1000 copies/ml (vs. ≥1000 copies/ml), for children admitted during the Option B+ period, compared to children admitted before the Option B+ period (Figure 4.4).

**Figure 4:**
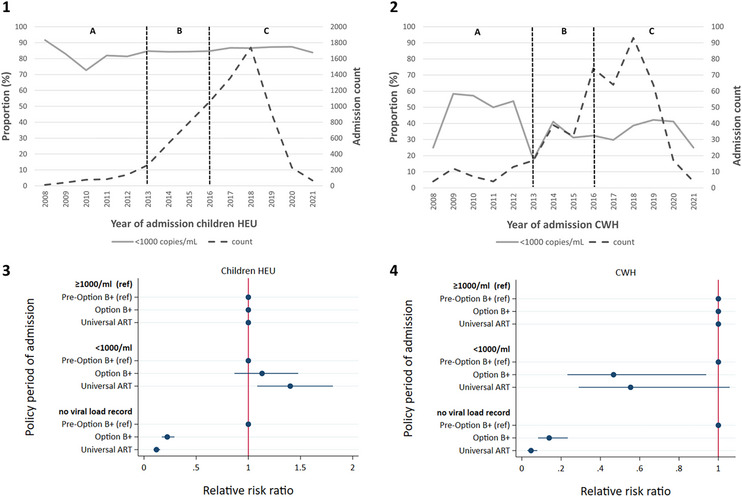
Maternal viral load. [1] and [2]—Total numbers of and trends in the proportion of infectious disease (lower respiratory tract infection, diarrhoea, meningitis and tuberculous meningitis) hospital admittees HEU [1] (*n* = 7306) or with HIV [2] (*n* = 444) whose mothers had a viral load of <1000 copies/ml during pregnancy (including only mothers with a viral load record). The vertical dotted lines demarcate different policy periods: A—Pre‐Option B+, B—Option B+, C—Universal ART; [3] and [4]—Plot of the relative risk ratios (with 95% confidence intervals) from multinomial logistic regression assessing the association of maternal viral load during pregnancy with policy period of hospital admission, for children HEU [3] (*N* = 8969) and CWH [4] (904), respectively. *We had no viral load record for 1663 (18.5%) of mothers to children HEU and 460 (51.0%) of CWH. ART, antiretroviral therapy; CWH, children with HIV; HEU, exposed to HIV and uninfected; ref, reference group.

Among hospitalised children HEU with mothers who had a CD4 count recorded, the proportion of mothers with CD4 count <350 cells/μl decreased from 90.6% in 2008 to 27.8% in 2021 (Figure 5.1). Among hospitalised CWH, the proportion of mothers with a CD4 count <350 cells/μl was 70.8% in 2008 and 75.0% in 2021, with a minimum of 41.2% in 2020 (Figure 5.2). Mothers of hospitalised children HEU were more likely to have a CD4 count ≥500 cells/μl (vs. <350 cells/μl), for children admitted during the Option B+ (RRR: 3.54; 95 CI%: 2.90‐4.31) and Universal ART periods (RRR: 4.74; 95 CI%: 3.92–5.73), compared to children admitted before the Option B+ period (Figure 5.3). Mothers of hospitalised CWH were less likely to have a CD4 count ≥500 cells/μl (vs. <350 cells/μl), for children admitted during the Universal ART period (RRR: 0.61; 95 CI%: 0.40–0.93), compared to children admitted before the Option B+ period (Figure 5.4).

**Figure 5:**
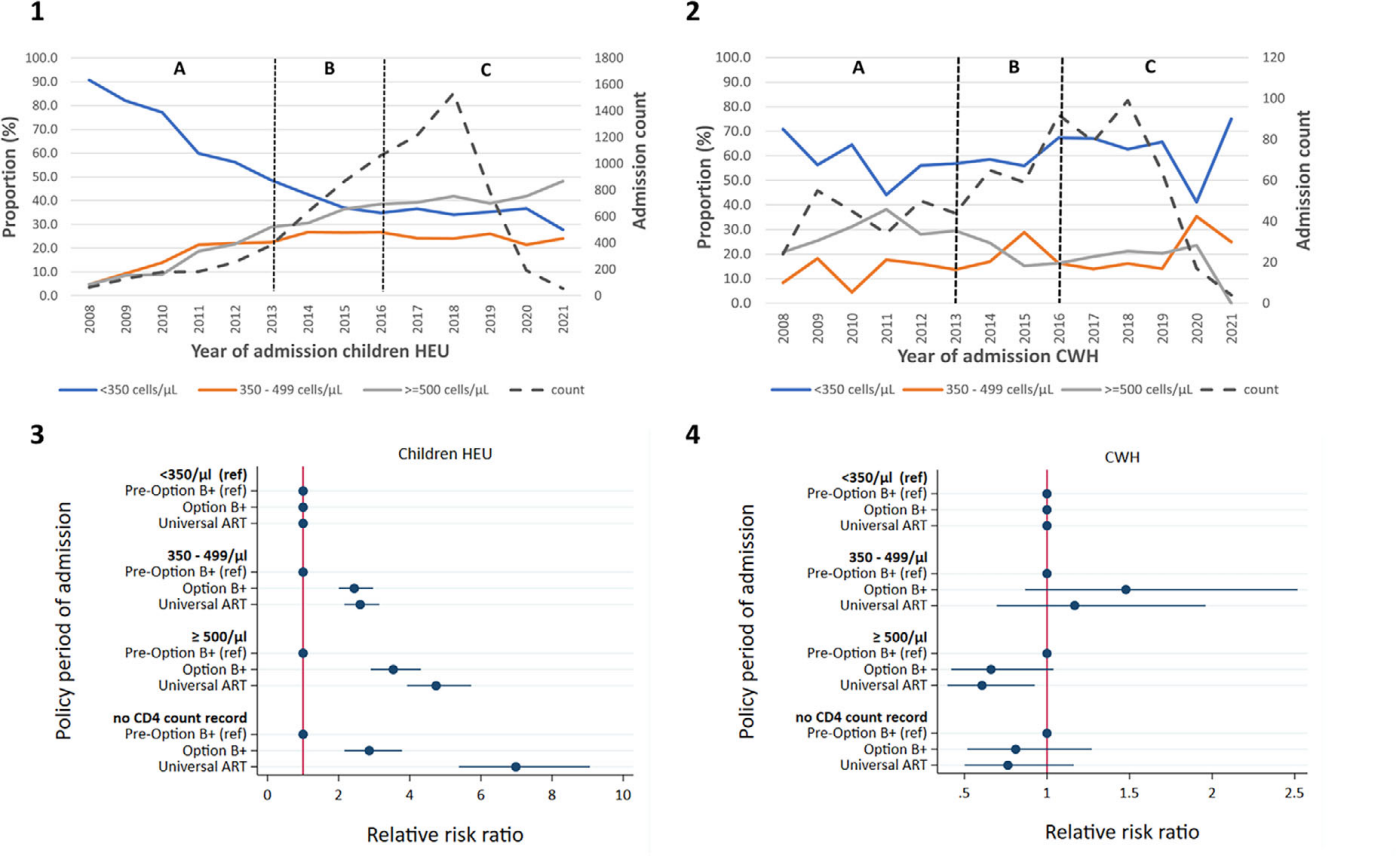
Maternal CD4 count. [1] and [2]—Total numbers of and trends in the proportion of infectious disease (lower respiratory tract infection, diarrhoea, meningitis and tuberculous meningitis) hospital admittees HEU [1] (*n* = 7523) or with HIV [2] (*n* = 731) whose mothers had CD4 count <350 cells/μl, 350–499 cells/μl or ≥500 cells/μl during pregnancy (including only mothers with a CD4 count record). The vertical dotted lines demarcate different policy periods: A—Pre‐Option B+, B—Option B+, C—Universal ART; [3] and [4]—Plot of the relative risk ratios (with 95% confidence intervals) from multinomial logistic regression assessing the association of mother CD4 count during pregnancy with policy period of hospital admission, for children HEU [3] (*n* = 8969) and CWH [4] (*n* = 904), respectively. *We had no CD4 count record for 1456 (16.2%) of mothers to children HEU and 173 (19.1%) of CWH. ART, antiretroviral therapy; CWH, children with HIV; HEU, exposed to HIV and uninfected; ref, reference group.

## 4 DISCUSSION

Since the scale‐up of VTP in SA from 2002, tremendous gains have been made in preventing and treating paediatric HIV acquisition [9, 27]. Our findings demonstrate how the HIV‐related profile of children hospitalised for infectious diseases has changed from 2008 to 2021 in the Western Cape, in the context of guideline amendments.

During the scale‐up of VTP, the stabilisation of antenatal HIV prevalence and reduction in vertical HIV transmission resulted in a progressive decline in the CWH population and consequent increase in the prevalence of children HEU, since 2004 [27]. Thembisa model estimates show a decrease in HIV prevalence, among children under the age 3 years in the Western Cape, from 1.0% in 2008 to 0.4% in 2021 [28]. While we do not have estimates for the prevalence of children HEU in the Western Cape, UNAIDS estimates of the national prevalence of children HEU (0–14 years) in SA increased from 11.8% in 2008 to 21.6% in 2018 [13]. Antenatal HIV prevalence in SA is higher than in the Western Cape (30.0% vs. 18.9% in 2019), therefore, we expect the prevalence of children HEU to be correspondingly lower in the Western Cape and that the average prevalence of children HEU between 2008 and 2018 (the birth cohorts of this analysis) would be lower than 17% [15]. These patterns are reflected in our analysis. During the 14‐year period under analysis, the proportion of CWH among infectious disease hospital admittees age ≤3 years dropped from 7.0% in 2008 to 1.3% in 2018. This corresponded with an increase in the proportion of children HEU from 14.1% in 2008 to 17.3% in 2018. With an average prevalence of children HEU over the study period of 17%, this suggests an over‐representation of hospitalised children HEU than in the general population. A study by Meyers et al. also showed a decreasing trend in the HIV prevalence among hospital admittees (0–14 years), although the HIV prevalence among hospital admittees that they reported for 2007 and 2010/11 (29.5% and 19.3%) was substantially higher than what we observed in 2008 and 2010, respectively [17]. The Meyers et al. study was conducted at a single large urban academic hospital in Gauteng province, which has a higher HIV prevalence among children than Western Cape, and results may not be generalizable to our province‐wide setting in the Western Cape.

The introduction of Option B+ has improved access to ART for all pregnant people living with HIV and dramatically reduced vertical HIV transmission [9]. In the Western Cape, between 2010 and 2013, Myer et al. observed a substantial increase in the proportion of pregnant people living with HIV entering antenatal care on ART, and initiating ART before delivery [29]. They also reported a substantial reduction in delays to antenatal ART initiation after CD4 eligibility criteria were removed during the Option B+ period. In our analysis, mothers of children admitted to hospital in the Option B+ and universal ART periods, compared to Pre‐Option B+, were significantly more likely to have been diagnosed with HIV before pregnancy versus during pregnancy and to have initiated ART before or during pregnancy versus at delivery/postnatally. As a result, the proportion of children exposed to maternal ART increased over time, with children admitted during universal ART versus Pre‐Option B+, having eight times higher odds of maternal ART exposure. Furthermore, with mothers initiating ART earlier, children admitted in the later periods, compared to Pre‐Option B+, were more likely to have been exposed to maternal ART for the first time at conception versus late gestation/postnatally. The apparent slight increase from 2016 onwards in the proportion who had no ART evidence among mothers with HIV may be due to better ascertainment of maternal HIV status in this period.

Throughout the study period, mothers of CWH at the time of infectious disease hospital admission predominantly had viral loads ≥1000 copies/ml near delivery, while the proportion of mothers of children HEU with viral loads <1000 copies/ml near delivery was >80% for all years except 2010. This is expected, given that the risk of vertical HIV transmission is increased with a higher maternal viral load, particularly ≥1000 copies/ml [30, 31].

As access to maternal ART improved over the years, and CD4 count was no longer recommended for monitoring persons living with HIV who are virally suppressed on ART, the proportion of mothers of children HEU who had CD4 count recorded reduced, as did the proportion with CD4 <350 cells/μl near delivery, suggesting that maternal ART has not only reduced vertical HIV transmission, but also improved maternal health, as expected. The reduction in maternal deaths by the time of child age 3 years supports this. We found that among CWH admittees, mothers predominantly had CD4 counts <350 cells/μl near delivery, corresponding with high viral loads observed in these mothers, suggesting that mothers not optimally sustaining ART in the context of a high coverage effective VTP programme remain especially vulnerable.

### 4.1 Strengths and limitations

The Western Cape Provincial Health Data Centre provided a novel opportunity to assess, province‐wide, the real‐world trends in HIV‐related characteristics of hospitalised children, using individual‐level longitudinal health service data. We were also able to classify HIV exposure and acquisition status of children at the time of hospitalisation, according to standardised certainty definitions [32], enabling better comparability with other studies that use the same definitions.

A limitation is that not all children were classified by HIV exposure and acquisition status with high certainty, particularly children HUU, of whom >90% were classified with low certainty (largely because Rapid point‐of‐care HIV results are not routinely digitised). It is possible that CWH may have been misclassified as HEU and children HEU as HUU. However, due to high rates of antenatal HIV testing within SA (>95%) [9], we are confident that most mothers living with HIV would have been identified during pregnancy, thereby limiting the misclassification of children exposed to HIV as unexposed. Additionally, of the children excluded from our analysis (*n* = 1370), 70% had mothers diagnosed with HIV postnatally. We may, therefore, have a slight underestimation of children HEU or with HIV in our sample.

Another limitation of our findings is the accuracy and completeness of ICD‐10 codes, particularly in the earlier years of the analysis. Our classification of hospital admissions due to infectious causes relied on ICD‐10 codes, which were previously shown to have poor reliability [33]. However, in more recent years, the implementation of a standardised discharge summary has improved ICD‐10 code completeness and accuracy [34], likely resulting in improved identification of infectious cause hospitalisations. As a result, a large proportion of admissions included in our analyses were from 2015 onwards. Furthermore, it is probable that in the earlier years, admission codes were captured more accurately in tertiary hospitals and among more severely sick children. In the Pre‐Option B+ period, >70% of admissions in our analyses were to tertiary hospitals (not shown), whereas >70% were to non‐tertiary hospitals during the universal ART period. It is possible that the Pre‐Option B+ cohort is more representative of severely ill children than the cohorts of children admitted in the later periods, potentially overestimating the prevalence of CWH, and other risk factors for severe disease, particularly low birth weight.

We did not include children born out of the province who relocated to and were hospitalised in the Western Cape. We also only considered four infectious diseases that cause substantial morbidity and mortality in children. Other childhood infections, including pulmonary tuberculosis, were not included in this analysis. Our results may, therefore, not be generalizable to all child infectious diseases burdening the healthcare system.

## 5 CONCLUSIONS

Temporal trends among children hospitalised with infectious diseases highlight the positive impact of VTP and increased ART access within SA. Whereas children of mothers with HIV were previously exposed to no or short‐duration maternal ART, in recent years, the majority were exposed to maternal ART, frequently from early gestation. There were fewer CWH and a higher proportion of children HEU in recent years.

However, the finding that at least one in six children hospitalised in recent years were HEU, of which up to 87% were exposed to maternal ART, highlights the need to consider HIV and ART exposure status, and not just child HIV prevalence, when assessing the impact of the HIV epidemic on child health services. Further research is needed to quantify the burden of infectious diseases on the health system that is due to higher risk among children HEU relative to children HUU and whether there is a need for HEU‐specific interventions in addition to interventions that improve the health and wellbeing of all children in resource‐limited settings.

## COMPETING INTERESTS

KA, EK, AB and M‐AD receive funding from ViiV Healthcare for an unrelated project.

## AUTHORS’ CONTRIBUTIONS

STdB, M‐AD, ALS, BE, SMI and HEJ conceptualised the research study. STdB managed the data with assistance from FP and insight from M‐AD, ALS, KA and EK. AB provided data engineering oversight within the Western Cape Provincial Health Data Centre. STdB conducted the data analyses and drafted the manuscript with subject matter expertise and/or scientific oversight from M‐AD, ALS, BE, SMI, HEJ and KA. All authors reviewed and approved the final manuscript.

## FUNDING

This work was supported by the University of Bristol's (i) Pro Vice‐Chancellor Research and Enterprise Strategic Research Fund and (ii) Global Challenge Research Fund Strategy funded by Research England; by the NIH Fogarty International Centre under grant K43TW010683 to ALS; by the Eunice Kennedy Shriver National Institute of Child Health and Human Development of the National Institutes of Health under grant R61HD103093 to ALS and M‐AD; by the Western Cape Department of Health to the Western Cape Provincial Health Data Centre; by the US National Institutes for Health under grants R01 HD080465 and U01 AI069924 to the Western Cape Provincial Health Data Centre; and the Bill and Melinda Gates Foundation under grants 1164272 and 1191327 to the Western Cape Provincial Health Data Centre.

## Supporting information


**Figure S1**: Flow diagram of mother‐infant pairs included in the cohort of children, born in the Western Cape (2008 – 2018), who had an infectious disease hospital admission (lower respiratory tract infection, diarrhoea, meningitis, tuberculous meningitis) by age three years.
**Figure S2: Infant exposure to maternal ART. (1)** ‐ Trend in the proportion of hospital admittees HEU or with HIV who were exposed to maternal ART and hospitalised with at least one of four infectious diseases (lower respiratory tract infection, diarrhoea, meningitis, tuberculous meningitis), by year. The vertical dotted lines demarcate different policy periods: A Pre‐Option B+, B ‐ Option B+, C ‐ Universal ART; **(2)** ‐ Plot of the odds ratios (with 95% confidence intervals) from logistic regression assessing the association of infant exposure to maternal ART with policy period of hospital admission. N = 9,873.
**Figure S3: Timing of earliest infant exposure to maternal ART. (1)** ‐ Trends in the proportion of hospital admittees' earliest exposure to maternal ART at different time points, among those who were exposed to maternal ART and hospitalised with at least one of four infectious diseases (lower respiratory tract infection, diarrhoea, meningitis, tuberculous meningitis), by year. The vertical dotted lines demarcate different policy periods: A Pre‐Option B+, B ‐ Option B+, C ‐ Universal ART; **(2)** ‐ Plot of the relative risk ratios (with 95% confidence intervals) from multinomial logistic regression assessing the association of timing of initial infant exposure to mother's ART start (relative to pregnancy and delivery) with policy period of hospital admission. N = 7,612.
**Table S1**: Simplified DECIPHER definitions for classification of children as HEU and HUU from routinely‐collected data.
**Table S2**: Count and proportion of hospital admissions per year
**Table S3**: Certainty of HIV exposure status in children born to women with and without HIV, in Western Cape, South Africa (2008‐2021), at hospitalisation.
**Table S4**: Number of mothers and children with evidence for maternal HIV, maternal ART, Infant exposure to maternal ART, maternal viral loads and maternal CD4 counts, among children HEU and with HIV.
**Table S5**: Descriptive statistics for Maternal HIV diagnosis and Maternal ART start across the three policy periods, for mothers who had evidence of an HIV diagnosis (N=9,873).

## Data Availability

The data that support the findings of this study are available from the Western Cape Provincial Health Data Centre, but restrictions apply to the availability of these data, which were used under license for the current study, and so are not publicly available. Data are available, however, from the corresponding author upon reasonable request and with permission of the Western Cape Provincial Health Data Centre.
