# Supplementary material for: Change in HIV‐related characteristics of children hospitalised with infectious diseases in Western Cape, South Africa, 2008–2021: a time trend analysis
Source: J Int AIDS Soc. 2023 Nov 1;26(Suppl 4):e26151. doi: 10.1002/jia2.26151 (PMC10618898; doi:10.1002/jia2.26151)
Supplement: Supplementary file 1 — Figure S1: Flow diagram of mother‐infant pairs included in the cohort of children, born in the Western Cape (2008 – 2018), who had an infectious disease hospital admission (lower respiratory tract infection, diarrhoea, meningitis, tuberculous meningitis) by age three years. Figure S2: Infant exposure to maternal ART. (1) ‐ Trend in the proportion of hospital admittees HEU or with HIV who were exposed to maternal ART and hospitalised with at least one of four infectious diseases (lower respiratory tract infection, diarrhoea, meningitis, tuberculous meningitis), by year. The vertical dotted lines demarcate different policy periods: A Pre‐Option B+, B ‐ Option B+, C ‐ Universal ART; (2) ‐ Plot of the odds ratios (with 95% confidence intervals) from logistic regression assessing the association of infant exposure to maternal ART with policy period of hospital admission. N = 9,873. Figure S3: Timing of earliest infant exposure to maternal ART. (1) ‐ Trends in the proportion of hospital admittees' earliest exposure to maternal ART at different time points, among those who were exposed to maternal ART and hospitalised with at least one of four infectious diseases (lower respiratory tract infection, diarrhoea, meningitis, tuberculous meningitis), by year. The vertical dotted lines demarcate different policy periods: A Pre‐Option B+, B ‐ Option B+, C ‐ Universal ART; (2) ‐ Plot of the relative risk ratios (with 95% confidence intervals) from multinomial logistic regression assessing the association of timing of initial infant exposure to mother's ART start (relative to pregnancy and delivery) with policy period of hospital admission. N = 7,612. Table S1: Simplified DECIPHER definitions for classification of children as HEU and HUU from routinely‐collected data. Table S2: Count and proportion of hospital admissions per year Table S3: Certainty of HIV exposure status in children born to women with and without HIV, in Western Cape, South Africa (2008‐2021), at hospitalisation. [file JIA2-26-e26151-s001.docx]

# Supplementary materials

# Study participants

Linked mother-child pairs with an electronically recorded infectious disease hospital admission (lower respiratory tract infection, diarrhoea, meningitis and tuberculous meningitis) by age three years, in the Western Cape Provincial Health Data Center

N=54,181

Excluded:

Data inconsistencies (e.g. child HIV-PCR negative after their first HIV evidence date); N=127

Mother not linkable (maternal data unknown); N=99

Mother diagnosed with HIV postnatally; N=946

Timing of infant HIV acquisition unknown; N=198

Total mother-infant pairs included in analysis:

N=52,811

Admission causes:

Lower respiratory tract infections: N=34,256 (64.9%)

Diarrhoea: N=19,157 (36.3%)

Meningitis: N=2,139 (4.0%)

Tuberculous meningitis: 293 (0.6%)

*Admissions could have been due to more than one cause

Admission age:

0 – 6 months: N=23,428 (44.4%)

>6 – 12 months: N=12,221 (23.1%)

>12 – 24 months: N=12,260 (23.2%)

>24 – 36 months: N =4,902 (9.28%)

***Supplementary Figure 1:*** *Flow diagram of mother-infant pairs included in the cohort of children, born in the Western Cape (2008 – 2018), who had an infectious disease hospital admission (lower respiratory tract infection, diarrhoea, meningitis, tuberculous meningitis) by age three years.*

# Classification of child HIV exposure and acquisition status

***Child HIV* acquisition *status***

- **With HIV**: child that has evidence of HIV before or at time of hospitalisation (before discharge date).
- **Without HIV:** if the child had no evidence of having HIV OR if the child’s first evidence of HIV was after hospital discharge and they had a negative HIV test result after hospital admission and before their date of first HIV evidence.
- **Unknown (children that seroconverted):** child’s date of first HIV evidence is after hospital discharge and they had evidence of a negative HIV test result before hospital admission or had never previously been tested for HIV.

***Child HIV exposure status (children without HIV)***

- **Unexposed to HIV and uninfected (HUU):** if the mother had no evidence of HIV OR the mother’s first evidence of HIV was more than 3 years post infant date of birth.
- **Exposed to HIV (antenatally) and uninfected (HEU):** if the mother’s first evidence of HIV was ≤ 10 weeks post infant date of birth.

# *HIV exposure and absence of child HIV* acquisition *certainty classifications*

We retrospectively classified **certainty of HIV exposure** **without HIV acquisition** at time of hospitalisation according to simplified DECIPHER definitions (Table 1). The detailed DECIPHER definitions have been published [1]. We used child HIV exposure status (defined above) to retrospectively classify children as HEU or HUU. Children with HIV (CWH) and those whose first HIV evidence date was after the timepoint of interest were excluded from classification. Certainty classification of children HEU was dependant on the timing of the child’s HIV testing while certainty classification of children HUU was dependant on the timing of both mother and child HIV testing. We extracted routine data (test type, date of test, and test result) on the **last negative** PCR or serology HIV test result for children that were tested for HIV, up until 31 December 2021, from the Western Cape Provincial Health Data Centre. Where a record of last maternal HIV negative test was available for mothers, it was extracted from the Western Cape Provincial Health Data Centre. We used these infant and maternal records to retrospectively classify children. Illustrations to help understand the DECHIPHER definitions are available in the supplementary materials of their published paper [1].

**Supplementary Table 1:** Simplified DECIPHER definitions for classification of children as HEU and HUU from routinely-collected data.

| **HEU – high certainty:** Child of a mother known to have HIV AND child *tested HIV-negative* (antibody or nucleic acid test) *at >6 weeks of age* AND at/after study outcome measurement |
| --- |
| **HEU – moderate certainty:** Child of a mother known to have HIV AND child *tested HIV-negative* (antibody or nucleic acid test) at least once at age ≥6 weeks but before study outcome measurement |
| **HEU – low certainty:** Child of a mother known to have HIV AND child tested *HIV-negative* by nucleic acid test at <6 weeks of age only |
| **Exposed to HIV - no certainty that child is HIV-uninfected:** Child of a mother known to have HIV AND child never tested for HIV |
| **HUU – high certainty:** Child of a *mother who tested HIV-negative* by any test type at/after the time of study outcome measurement AND child *tested HIV-negative* by any test type at least once at any time |
| **HUU – moderate certainty:** Child whose *mother’s test* meets high certainty criteria but child has never tested for HIV |
| **HUU – low certainty:** Child whose *mother tested HIV-negative* during pregnancy but before study outcome measurement OR *mother* has no record of a HIV-negative test and no evidence of HIV |

HEU: exposed to HIV and uninfected; HUU: unexposed to HIV and uninfected

**Results**

# *Hospital admissions:*

**Supplementary Table 2:** Count and proportion of hospital admissions per year

| Admission year | Frequency (N) | Percent (%) |
| --- | --- | --- |
| 2008 | 512 | 1.0 |
| 2009 | 1,088 | 2.1 |
| 2010 | 1,338 | 2.5 |
| 2011 | 1,400 | 2.7 |
| 2012 | 1,814 | 3.4 |
| 2013 | 2,878 | 5.5 |
| 2014 | 3,855 | 7.3 |
| 2015 | 5,590 | 10.6 |
| 2016 | 6,786 | 12.9 |
| 2017 | 8,238 | 15.6 |
| 2018 | 11,176 | 21.2 |
| 2019 | 6,276 | 11.9 |
| 2020 | 1,406 | 2.7 |
| 2021 | 454 | 0.9 |
| Total | 52,811 | - |

***Infant HIV exposure and* acquisition *status:***

**Supplementary Table 3:** Certainty of HIV exposure status in children born to women with and without HIV, in Western Cape, South Africa (2008-2021), at hospitalisation

|  | Prior to Option B+ | During Option B+ | Universal ART | Total |
| --- | --- | --- | --- | --- |
| **CHILDREN HEU (N =8,969)** |  |  |  |  |
| High certainty N (%) | 591 (57.6) | 1,765 (63.0) | 2,698 (52.5) | 5,054 (56.4) |
| Moderate certainty N (%) | 218 (21.3) | 738 (26.3) | 2,037 (39.6) | 2,993 (33.4) |
| Low certainty N (%) | 88 (8.6) | 181 (6.5) | 308 (6.0) | 577 (6.4) |
| No certainty N (%) | 128 (12.5) | 118 (4.2) | 99 (1.9) | 345 (3.9) |
| **CHILDREN HUU (N = 42,864)** | |  |  |  |
| High certainty N (%) | 324 (5.5) | 472 (3.7) | 492 (2.1) | 1,288 (3.0) |
| Moderate certainty N (%) | 398 (6.8) | 632 (4.9) | 782 (3.3) | 1,812 (4.2) |
| Low certainty N (%) | 5,177 (87.8) | 11,838 (91.5) | 22,749 (94.7) | 39,764 (92.8) |

ART: Antiretroviral therapy; HEU: exposed to HIV and uninfected; HUU: unexposed to HIV and uninfected

***HIV-related characteristics:***

**Supplementary Table 4:** Number of mothers and children with evidence for maternal HIV, maternal ART, Infant exposure to maternal ART, maternal viral loads and maternal CD4 counts, among children HEU and with HIV

| HIV-related characteristic | Total | Children HEU | Children with HIV |
| --- | --- | --- | --- |
| **Maternal HIV** | **N=9,947** | **N=8,969** | **N=978** |
| Yes | 9,873 (99.3) | 8,969 (100.0) | 904 (92.4) |
| No evidence | 74 (0.7) |  | 74 (7.6) |
| **Maternal ART - N (%)** | **N= 9,873** | **N= 8,969** | **N=904** |
| Yes | 9,391 (95.1) | 8,576 (95.6) | 815 (90.2) |
| No evidence | 482 (4.9) | 393 (4.4) | 89 (9.9) |
| **Infant exposure to maternal ART – N (%)** | **N= 9,873** | **N= 8,969** | **N=904** |
| Yes | 7,612 (77.1) | 7,186 (80.1) | 426 (47.1) |
| No evidence | 2,261(22.9) | 1,783 (19.9) | 478 (52.9) |
| **Maternal viral load record – N (%)** | **N= 9,873** | **N= 8,969** | **N=904** |
| Yes | 7,750 (78.5) | 7,306 (81.5) | 444 (49.1) |
| No | 2,123 (21.5) | 1,663 (18.5) | 460 (51.0) |
| **Maternal CD4 count record – N (%)** | **N= 9,873** | **N= 8,969** | **N=904** |
| Yes | 8,244 (83.5) | 7,513 (83.8) | 731 (80.9) |
| No | 1,629 (16.5) | 1,456 (16.2) | 173 (19.1) |

ART: Antiretroviral therapy; HEU: exposed to HIV and uninfected

***Maternal HIV diagnosis and ART start time:***

**Supplementary Table 5:** Descriptive statistics for Maternal HIV diagnosis and Maternal ART start across the three policy periods, for mothers who had evidence of an HIV diagnosis (N=9,873)

| HIV-related characteristic | Total | Pre-Option B+ | Option B+ | Universal ART |
| --- | --- | --- | --- | --- |
| **Maternal HIV diagnosis** |  |  |  |  |
| Before pregnancy | 5,964 (60.4) | 550 (42.3) | 1,730 (56.3) | 3,684 (67.0) |
| During pregnancy | 3,370 (34.1) | 552 (42.4) | 1,163 (37.8) | 1,655 (30.1) |
| Delivery/Postnatally | 539 (5.5) | 199 (15.3) | 182 (5.9) | 158 (2.9) |
| **Maternal ART start** |  |  |  |  |
| Before pregnancy | 4,342 (44.0) | 238 (18.3) | 1,126 (36.6) | 2,978 (54.2) |
| During pregnancy | 3,520 (35.7) | 262 (20.1) | 1,304 (42.4) | 1,954 (35.6) |
| Delivery/Postnatally | 1,529 (15.5) | 626 (48.1) | 524 (17.0) | 379 (6.7) |
| No evidence | 482 (4.9) | 175 (13.5) | 121 (3.9) | 186 (3.4) |

# ART: Antiretroviral therapy

***Infant Exposure to maternal ART:***


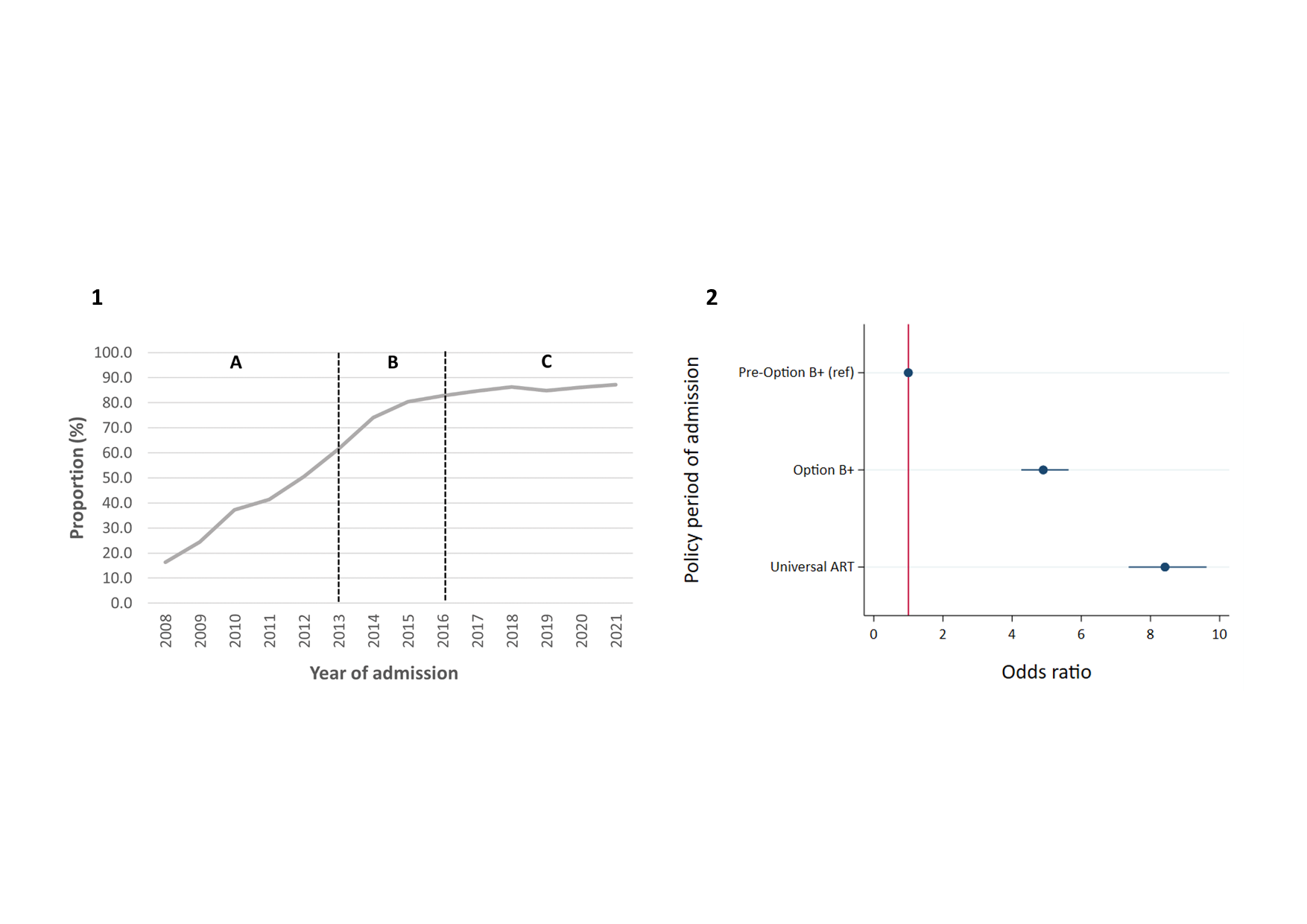


**Supplementary Figure 2: Infant exposure to maternal ART.**  **(1)** - Trend in the proportion of hospital admittees HEU or with HIV who were exposed to maternal ART and hospitalised with at least one of four infectious diseases (lower respiratory tract infection, diarrhoea, meningitis, tuberculous meningitis), by year. The vertical dotted lines demarcate different policy periods: A – Pre-Option B+, B - Option B+, C - Universal ART; **(2)** - Plot of the odds ratios (with 95% confidence intervals) from logistic regression assessing the association of infant exposure to maternal ART with policy period of hospital admission. N = 9,873.

ART: antiretroviral therapy; CWH: children with HIV; HEU: exposed to HIV and uninfected; ref: reference group


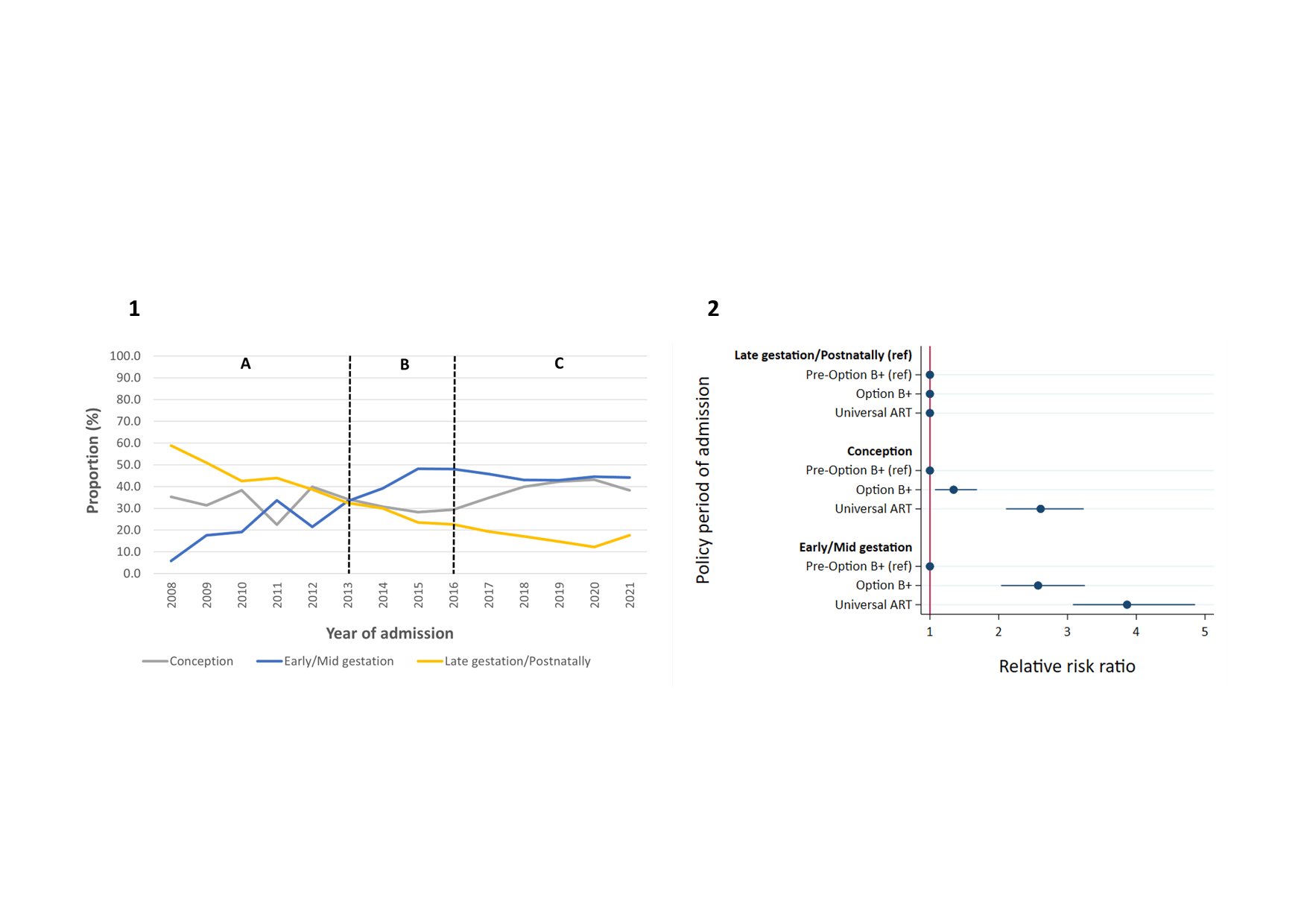


**Supplementary Figure 3: Timing of earliest infant exposure to maternal ART. (1)** - Trends in the proportion of hospital admittees’ earliest exposure to maternal ART at different time points, among those who were exposed to maternal ART and hospitalised with at least one of four infectious diseases (lower respiratory tract infection, diarrhoea, meningitis, tuberculous meningitis), by year. The vertical dotted lines demarcate different policy periods: A – Pre-Option B+, B - Option B+, C - Universal ART; **(2)** - Plot of the relative risk ratios (with 95% confidence intervals) from multinomial logistic regression assessing the association of timing of initial infant exposure to mother’s ART start (relative to pregnancy and delivery) with policy period of hospital admission. N = 7,612.

ART: Antiretroviral therapy; mid: middle, ref: refence group

#

# References:

1. Slogrove AL, Burmen B, Davies M-A, et al. Standardized Definitions of In Utero Human Immunodeficiency Virus and Antiretroviral Drug Exposure Among Children. Clin Infect Dis **2021**; :1–9.
